# Fracture of the greater trochanter of the femur in 17 cats: imaging, clinical features and concurrent injuries

**DOI:** 10.1177/1098612X241305918

**Published:** 2025-04-03

**Authors:** Genziana Nurra, Mariette Pilot, Beatrice Grek-Fritzner, Mario Coppola, James Michael Grierson, Sorrel Langley-Hobbs

**Affiliations:** 1 Dick White Referrals, Six Mile Bottom, Cambridgeshire, UK; 2 Small Animal Referral Hospital Langford, University of Bristol, Bristol, UK; 3 Fitzpatrick Referrals, Godalming, Surrey, UK; 4 Anderson Moores Veterinary Specialists, Winchester, Hampshire, UK

**Keywords:** Concurrent orthopaedic injuries, greater trochanteric fracture, radiograph, proximal femur

## Abstract

**Objectives:**

The objectives of this study were to evaluate the population, concurrent injuries, best diagnostic radiographic projection, management and short-term outcome of cats presenting with a fracture of the greater trochanter.

**Methods:**

Medical records, imaging studies, methods of repair, complications and short-term outcome of cats that presented with a fracture of the greater trochanter were retrospectively reviewed at two referral institutions. Radiographs were evaluated using a quantitative scoring system. Short-term outcome (>3–6 months) and complications were determined at the follow-up appointment.

**Results:**

Seventeen cats were included. The mean age was 10.5 months (range 3–16). All 17 cats exhibited concurrent orthopaedic injuries, with 5/17 (29%) presenting with multiple orthopaedic injuries. Coxofemoral luxation was the most common, representing more than half (58.8%) of the concurrent orthopaedic injuries identified, followed by pelvic/sacral fractures (52.9%). Further orthopaedic injuries such as sacroiliac luxation and femoral neck fractures were the least common and were present in 11.7% of the studied population. Greater trochanteric fractures were most easily identified on the ventrodorsal radiographic projection of the pelvis, with increased accuracy in the frog-leg ventrodorsal view. Most cats (94.4%) were managed surgically using two or three Kirschner (K)-wires and a tension band wire. Short-term follow-up was available for all 17 cats at a mean of 6.3 months (range 3–8). Major complications were seen in 4/17 (23.5%) patients. These included mild lameness and persistent discomfort due to periosteal reaction, which resolved following surgical or medical intervention. Short-term mobility was considered good in 58.8% of cats, acceptable in 29.5% and poor in the remaining 11.7%.

**Conclusions and relevance:**

Coxofemoral luxation was the most prevalent concurrent orthopaedic injury (58.8%). The majority of the greater trochanteric fractures (94.4%) were managed surgically with K-wires and a tension band wire. Ventrodorsal and specifically frog-leg radiographic projections of the pelvis enhance the diagnosis of greater trochanteric fractures. Specific outcomes of greater trochanteric fractures are uncertain because of the high occurrence of concurrent orthopaedic injuries.

## Introduction

Proximal femoral fractures are common in both feline and canine populations and account for approximately 25% of femoral fractures. They are especially prevalent in young cats and are often associated with vehicular trauma.^1,2^ Fractures or avulsion of the greater trochanter are a specific subset of proximal femoral fractures. These are typically described in dogs as Salter–Harris type 1 fractures,^3^ occurring as a physeal separation between the greater trochanter and the proximal femur. They are often accompanied by proximal displacement of the fragment due to the tensile forces of the gluteal musculature.^3,4^ Immature animals are more susceptible to greater trochanteric fractures because of the vulnerability of growth plates.^5^ Fractures of the greater trochanter have been reported in cats less than 1 year of age, before growth plate closure, which occurs between 38 and 42 weeks of age in intact cats.^5,6^ Greater trochanteric fractures are commonly associated with concurrent orthopaedic injuries, which include coxofemoral luxation and fracture of the ipsilateral capital femoral physis or femoral neck.^3,7,8^ Definitive diagnosis is often based on radiographic findings although greater trochanteric fractures are not always easily detectable with this imaging modality.^9^

The management of greater trochanteric fractures in dogs has historically been influenced by the severity of fragment displacement. If displacement is minimal, conservative treatment with strict cage rest for 3–4 weeks can be considered as it may be sufficient to allow acceptable healing without loss of function.^3,4^ Surgical treatment is indicated when the greater trochanter is considerably displaced.^3^ In the veterinary literature, there is currently no established classification system or specific criteria to objectively assess the degree of fracture displacement. In this study, however, we considered partial displacement to have occurred when the separation between the fragments was <5 mm and considerable displacement to have occurred when the separation was >5 mm.

Primary fixation in dogs and cats has been reported to result in a good functional outcome, relying on the restoration of the gluteal muscles and restoring the normal biomechanics of the hip joint.^3,7^

To effectively convert tensile into compressive forces along the fracture line, fractures of the greater trochanter are often stabilised by placing one or two pins and a tension band wire.^4,7,10^ Compression across the greater trochanteric physis can result in early closure. Two related experimental studies in dogs evaluated the effect of premature closure of the greater trochanteric femoral physis.^11,12^ Both studies found that premature closure of the greater trochanteric physis did not affect the longitudinal length of the femur, although subtle changes in the conformation of the proximal femur may alter hip joint biomechanics and predispose dogs to coxofemoral degenerative joint disease. There is limited information available regarding the management and outcome of feline greater trochanteric fracture.^9^

The objectives of this study were to: (1) describe greater trochanteric fractures and the commonly associated concurrent injuries encountered in the feline population; (2) determine the best radiographic position for fracture detection; (3) describe management options; and (4) report complications and short-term outcomes.

## Materials and methods

Medical records and diagnostic imaging findings of cats that presented for the management of greater trochanteric fractures at two referral institutions (Fitzpatrick Referrals and Langford Vets) from 1 January 2009 to 1 January 2023 were reviewed. Cases were excluded if medical records were incomplete or follow-up, which included clinical examination and radiographs when available, was less than 3 months. Recorded data included: signalment, cause of injury, imaging type and imaging findings, concurrent injuries, stabilisation methods, complications and follow-up. Radiographic evaluation was performed at 4–8 weeks after surgery (depending on the surgeon’s preference) to assess the progression of fracture site healing and implant position. This included assessment of lateral projections of the affected femur and ventrodorsal radiographs of the pelvis.

Complications and subjective clinical outcomes were classified as described by Cook et al.^13^ Complications were categorised as either minor or major. Minor complications were defined as those not requiring additional surgical or medical treatment to resolve (eg, seroma or bruising). Major complications were defined as those requiring additional medical or surgical intervention for resolution (eg, infection, or other issues necessitating removal of the implant). Where available in the clinical records, lameness evaluations within 6 months after surgery were retrieved. Short-term clinical outcome (assessed between 3 and 6 months) and complications were determined from the patient records during follow-up appointments. Outcome was classified based on Cook et al.^13^

- Good outcome implies that the patient returned to preinjury levels of activity with no reported lameness or requirement for medication to achieve this level of function at the last evaluated time point.
- Acceptable function was defined as restoration to near-normal preinjury levels of activity for the patient where a mild lameness that was not considered to reduce the quality of life or require medication to achieve persisted.
- Poor function encompassed all other outcomes, including failure of surgical repair, muscle contracture, moderate or severe lameness, or euthanasia.

## Results

Seventeen cats met the inclusion criteria (Table 1). They had a mean age of 10.5 months (range 3–16). Ten cats were male (seven castrated, three entire), seven cats were female (four spayed, three entire). All 17 cats included in this study had access to outdoor space. Just over half of the cats (9/17; 52.9%) had confirmed witnessed vehicular trauma. The exact cause of the fractures in the eight remaining cats was unknown; however, given the presentation, we presumed that a traumatic event had also occurred in these cats.

**Table 1:** Clinical data, type of concurrent orthopaedic injuries, imaging modality and surgical management of 17 cats with greater trochanteric fracture

Case	Age (months)	Sex	Breed	Concurrent orthopaedic injuries	Preoperative imaging	Method of repair of the greater trochanteric fracture
1	12	ME	DSH	• Bilateral coxofemoral luxation	Radiographs	Two K-wires and tension band
2	8	FN	DSH	• Ischial tuberosity fracture • Ipsilateral pubic and sacral fractures • Ipsilateral SI luxation	CT	Two K-wires and tension band
3	15	FE	DSH	• Ipsilateral comminuted ischial fractures • Multiple bilateral comminuted pubic fractures • Bilateral sacroiliac fractures	CT	None – conservative treatment
4	9	FE	DSH	• Ipsilateral femoral neck fracture	Radiographs	Two K-wires and tension band
5	8	MN	DLH	• Contralateral transverse ischial fracture • Ipsilateral transverse ilial fracture • Ipsilateral coxofemoral luxation	CT + radiographs	Two K-wires and tension band
6	9	MN	DSH	• Ipsilateral coxofemoral luxation • Hip dysplasia	Radiographs	Two K-wires and tension band
7	12	ME	DSH	• Bilateral coxofemoral luxation	Radiographs	Two K-wires and tension band
8	12	MN	DLH	• Ipsilateral coxofemoral luxation • Contralateral ischial tuberosity fracture	Radiographs	Two K wires and tension band
9	9	ME	DSH	• Contralateral transverse ischial fracture • Ipsilateral femoral neck fracture	Radiographs	Two K-wires and tension band
10	3	FE	DSH	• Ipsilateral femoral neck fracture	CT + radiographs	Two K-wires and tension band
11	12	FN	DSH	• Ipsilateral ischial tuberosity fracture • Distal diaphyseal tibial/fibular fracture	Radiographs	Two K-wires and tension band
12	6	FN	DSH	• Ipsilateral coxofemoral luxation • Ipsilateral femoral head fracture	Radiographs	Three K-wires and tension band
13	12	MN	BSH	• Ipsilateral coxofemoral luxation • S-H type 3 tibial and malleolar fracture	Radiographs	Three K-wires and tension band
14	7	FN	Bengal	• Pubic fracture • Ipsilateral SI luxation • Ipsilateral proximal ulnar fracture	Radiographs	Two K-wires and tension band
15	14	MN	DSH	• Ipsilateral coxofemoral luxation	Radiographs	Three K-wires and tension band
16	16	MN	DSH	• Bilateral coxofemoral luxation	Radiographs	Two K-wires and tension band
17	5	MN	DSH	• Ipsilateral coxofemoral luxation	Radiographs	Two K-wires and tension band

BSH = British Shorthair; DLH = domestic longhair; DSH = domestic shorthair; FE = female entire; FN = female neutered; ME = male entire; MN = male neutered; SI = sacroiliac; S-H = Salter-Harris

**Figure 1:**
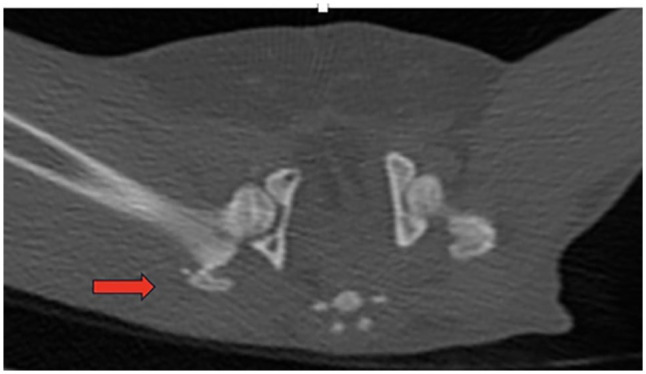
Case 2. Transverse CT image of the pelvis showing a greater trochanteric fracture (red arrow)

Radiography alone was performed in 13/17 cats (76.4%), whereas two of the 17 (11.8%) had a CT scan performed along with radiography. Two cats had CT as the only imaging modality performed (11.8%) (Figure 1).

Five of the 15 cats with radiographic images were referred from the primary veterinarian with three radiographic projections available (a mediolateral view of the affected femur, a ‘frog-leg’ ventrodorsal view of the pelvis and a ventrodorsal view of the pelvis with extended legs). The remaining 10 cats had two radiographic projections available: all 10 had a mediolateral projection, while a ventrodorsal frog-leg projection was available for six cats and a ventrodorsal extended-leg projection for four.

**Figure 2:**
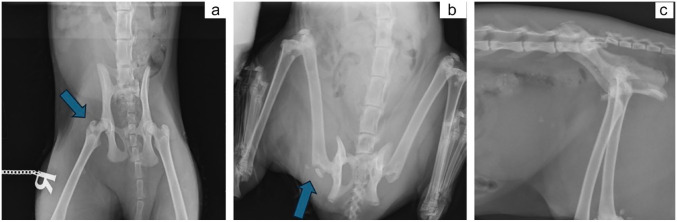
Case 14. (a) Extended-leg ventrodorsal radiograph of the pelvis showing a fracture of the right greater trochanter (blue arrow). This radiographic view was given a score of 2, as the fracture line is partially obscured by the neck of the femur. The cat also has ipsilateral sacroiliac luxation and a pubic fracture. (b) The same cat had a frog-leg ventrodorsal radiograph taken, which improved the radiographic accuracy for detecting the greater trochanteric fracture (blue arrow) and scored as 1. (c) Lateral radiographic view of the caudal abdomen and pelvis of the same cat. The greater trochanteric fracture in this view is very difficult to detect given the superimposition of the hemipelves, with the wings of the ilium obscuring the greater trochanteric fracture, and was given a score of 3

The radiographs were retrospectively evaluated by four surgeons (two board-certified surgeons, one board-eligible surgeon and one surgery resident). A quantitative scoring system was applied to each image in the radiographic series, based on the semi-quantitative scoring system used for diagnosing maxillofacial trauma in cats and dogs.^14^ Scoring (1–3) was defined as ‘1’ when the greater trochanteric fracture line was clear with the fragment separate from other bones/not obscured, ‘2’ when the greater trochanteric fracture line was partially obscured by bone and ‘3’ when the greater trochanteric fracture line was completely obscured by the superimposed bone (Figure 2). All images were evaluated by the four observers and image scoring was performed by consensus without reference to the medical records of these animals. Each observer first evaluated the scores independently before agreeing on the final consensus.

**Figure 3:**
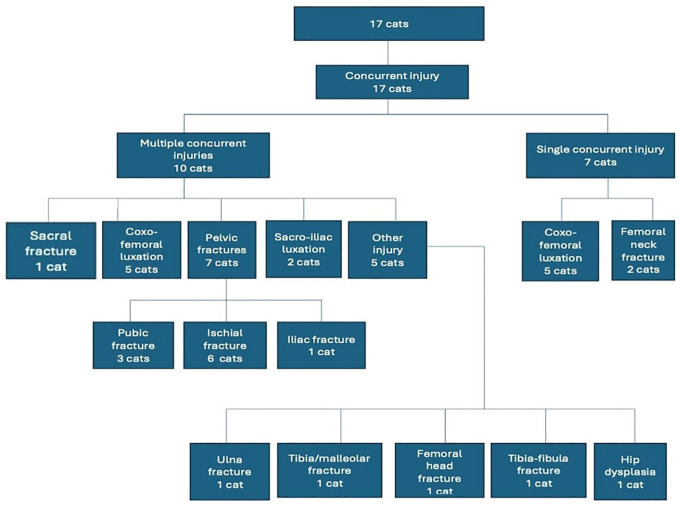
Flow chart illustrating the type and numbers of concurrent orthopaedic injuries associated with greater trochanteric fracture in 17 cats

All 17 cats in this study presented with concurrent orthopaedic injury and 10/17 cats had multiple concurrent injuries, which are outlined in Figure 3. Coxofemoral luxation was present in 10/17 cats and pelvic/sacral injury in 9/17 cats, including 3/17 pubic fractures, 6/17 ischial fractures, 1/17 ilial fracture, 1/17 sacral fracture and 2/17 sacroiliac fractures/luxation. Other types of injury/disease were identified in 7/17 and included femoral neck fractures in 2/17 and hip dysplasia, distal diaphyseal tibial/fibular fracture, ipsilateral femoral head fracture, Salter–Harris type 3 fracture of the distal tibia and malleolar fracture, and proximal ulnar fracture, each in 1/17 cats. A single concurrent injury was documented in 7/17 cats, comprising coxofemoral luxation in 5/17 and femoral neck fracture in 2/17 cats. Two different concurrent injuries were documented in 7/17 cats and three different concurrent injuries were documented in 3/17 cats.

## Management of the greater trochanteric fracture

Surgical repair of the greater trochanteric fracture was performed in 16/17 (94.4%) cats. The surgical approach to the greater trochanter was performed as previously described via a craniolateral approach to the hip.^15 –17^

**Figure 4:**
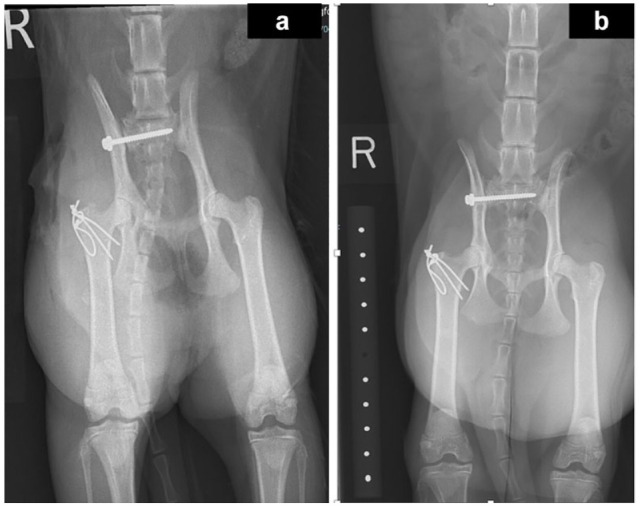
Case 2. (a) Immediate postoperative ventrodorsal extended-leg radiograph of the pelvis showing the greater trochanteric fracture surgical repair using two K-wires and a tension band. The cat also underwent surgical stabilisation of an ipsilateral sacroiliac luxation. (b) Repeat radiograph performed 6 weeks postoperatively showing progressive healing of the fracture site

Pins/Kirschner (K)-wires and tension band wire fixation was performed in all 16 cats that underwent surgery (Figure 4). Either two or three K-wires (in 14/16 and 3/16 cats, respectively) were placed across the greater trochanter into the proximal femur in a lateromedial direction with the aim to exit through the medial cortex of the proximal femur. A figure-of-eight stainless steel wire was placed through a predrilled hole in the proximal femur and around the preplaced pins to form the pin and tension band wire construct. The pin/K-wire diameter was recorded in 14/16 cats and varied from 0.8 mm to 1.4 mm. The orthopaedic wire diameter/gauge varied from 0.8 mm to 1.0 mm. In one cat, the greater trochanteric fracture was not treated surgically, but surgical stabilisation of the concurrent bilateral sacroiliac luxation with a lag screw was performed.

## Quantitative scoring system

Each of the three radiographic views (mediolateral view of the affected femur, frog-leg ventrodorsal view of the pelvis, ventrodorsal view of the pelvis with extended legs) were evaluated individually for ease of identifying the greater trochanteric fracture.

The fracture was most easily identified on the frog-leg ventrodorsal radiographic projection of the pelvis (8/11 cases) vs mediolateral projections (3/15 cases). Greater trochanteric fracture was most easily identified only in 6/9 cases on the ventrodorsal extended-leg view of the pelvis (Table 2). Fracture of the greater trochanter was easily identifiable in all four cats that had CT scans.

**Table 2:** Scoring system demonstrating the ability to identify fractures of the greater trochanter in cats across three radiographic views

Score	Ventrodorsal extended view	Ventrodorsal frog-leg view	Mediolateral view
1	6 cats	8 cats	3 cats
2	3 cats	3 cats	6 cats
3	0 cats	0 cats	6 cats

## Follow-up

Short-term follow-up was available for all 17 cats at a mean of 6.3 months (range 3–8) postoperatively. The majority of cats (10/17; 58.8%) had no lameness and healing of the fracture site was documented on the radiographs with all implants in place and unchanged in position. Major complications, not directly related to the greater trochanteric fracture or fracture repair, which required medical intervention were recorded in 3/17 cats (17.6%). Two cats had mild intermittent lameness (graded 1/5), which resolved following cage rest and administration of non-steroidal anti-inflammatory drugs. In the third cat, mineralisation at the tendon attachment of the abductor muscle group to the femur resulted in mild lameness, graded as 2/5. This patient underwent shockwave therapy. Data determining functional outcome in this specific case were not available.

A major complication, potentially related to the greater trochanteric fracture repair, which required surgical intervention was recorded in one of the 17 cats (5.8%). In this case, the implants used to repair the greater trochanteric fracture were removed 7 months postoperatively as the cat experienced persistent discomfort and lameness. Radiographs of this cat documented a region of decreased bone opacity affecting the proximal right femoral metaphysis and femoral neck with associated periosteal new bone formation. Biopsy of the periosteal proliferation and the medullary canal were obtained during surgery and histopathology was suggestive of remodelling periosteal reaction. A follow-up appointment at 5 weeks following revision surgery for implant removal reported that the clinical signs had resolved.

One cat underwent surgical revision 6 weeks after surgery following failure of the transarticular pin used to repair a concurrent coxofemoral luxation. While the cat had reoccurrence of hip luxation, the pin and tension band wire used to stabilise the greater trochanteric fracture remained stable and in place. Furthermore, radiographs documented progression of healing at the greater trochanteric fracture site. This case was not included in the complications category. The cat underwent surgical revision consisting of femoral head and neck excision.

Short-term clinical outcome (>3–6 months), which was based on the evaluation of the radiographs, when available, and the orthopaedic examination, was considered good in 10/17 (58.8%) of cats, acceptable in 2/17 (29.5%) and poor in the remaining 2/17 (11.7%).

## Discussion

Fracture of the greater trochanter of the femur is an uncommon injury^18^ but must be considered when evaluating young cats with acute hindlimb lameness. The results of this study indicate that, if radiography is used, ventrodorsal frog-leg radiographic projections of the pelvis increase the likelihood of identifying greater trochanteric fractures, and therefore the accuracy of diagnosis. Greater trochanteric fractures were easily identifiable in all cases where CT scanning was performed.

Similar to the existing literature, which reports a mean age range of 5–9 months for fracture of the greater trochanter,^8,9,19^ the affected demographic observed in this study was young, with a mean age of 10.5 months. The slightly older average age may be explained by neutering and its effect on growth plate closure. In our study, 11/17 cats were neutered. As oestrogen and testosterone facilitate cartilage maturation in the growth plate, a reduction in these hormone levels through neutering can lead to a delay in physeal cartilage closure, allowing physeal fractures to occur in more mature cats.^20^

To minimise misdiagnosis, radiographs of the pelvis in a frog-leg position can be helpful to increase visualisation of capital physeal fractures.^16^ Difficulty in identifying greater trochanteric fractures in cats has previously been reported.^9^ The results of the present study indicate that the ventrodorsal projection of the pelvis was superior to mediolateral views for identifying greater trochanteric fractures. When evaluating for a potential proximal femoral fracture, it has been recommended that orthogonal pelvic radiographs be taken, including lateral, extended-leg ventrodorsal and frog-leg ventrodorsal projections.^9^ However, in cats with proximal physeal fractures, the extended-leg ventrodorsal radiograph of the hips may not consistently show obvious abnormalities. Furthermore, a more recent study documented that ventrodorsal frog-leg radiographs were more accurate in the diagnosis of slipped capital femoral epiphyses than ventrodorsal extended-leg projections when evaluating the S-sign.^21^ Mediolateral views alone may lead to misdiagnosis as the avulsed fragment of the greater trochanter can go undetected because of superimposition of the femoral neck and the ilial body.^9^

CT was performed for further assessment and surgical planning in only a few cases reviewed in this study, but the greater trochanteric fracture was evident in every one of these cases. CT has been confirmed to improve the accuracy of the diagnosis of other orthopaedic diseases such as humeral intracondylar fissure^22^ and mandibular fracture in cats.^14^ Furthermore, it has been demonstrated to be a valuable diagnostic method for the evaluation of the proximal region of the femur, particularly in cases in which subtle comminution, articular involvement or the presence of a fissure is suspected.^23^ High-detail digital radiographs and advanced imaging technology, such as a CT scan, were not commonly used in earlier studies.^2,8,12,16,19,24,25^ These current techniques could lead to increased diagnosis of greater trochanteric fractures in cats compared with previous radiographic techniques. Furthermore, in the present study, radiographs were evaluated by board-certified radiologists and surgery specialists, specifically looking for these fractures, and thereby increasing the likelihood of detection.

Greater trochanteric fractures in cats have been reported alongside coxofemoral luxation,^9^ femoral neck or femoral capital physeal fractures.^8^ None of the cats included in this study presented with a greater trochanteric fracture as their sole orthopaedic concern, reflecting the traumatic nature of their injury. This study found that a larger proportion of cats sustained multiple concurrent orthopaedic injuries, rather than a single concurrent orthopaedic injury and that coxofemoral luxation and pelvic/sacral injury were the most common concurrent injury types identified.

Surgical treatment with primary fixation has been reported as the treatment of choice when proximal displacement of the greater trochanter fragment occurs. This is because it preserves the normal anatomy by restoring gluteal muscle function and the normal biomechanics of the hip joint.^3,9^ Stabilisation techniques described in the literature for canine patients include pins and a tension band wire, lag screw augmented by an antirotational K-wire, and a bone plate.^3,24,26^ The majority of cases reviewed in this study underwent surgical reduction of the greater trochanteric fracture with pins and a tension band wire. Conservative management of the greater trochanteric fracture was used in one cat where the fracture was only minimally displaced. However, the same cat underwent surgery to repair concurrent bilateral sacroiliac luxation. Short-term outcome for this cat was good, with a complete return to function. Given that there was only a single case where the greater trochanteric fracture was treated conservatively, it was not possible to draw any conclusions about the benefits of surgical versus non-surgical management of this injury.

This study has some limitations. Due to the uncommon nature of this condition, the study population size was limited (n = 17). This is a retrospective study which could lead to incomplete data and missing complications. Furthermore, due to the multicentre nature of the data, imaging modalities and surgical interventions varied based on surgeon preference, which may influence outcomes. Separating the effect of the greater trochanteric fractures from the effect of other concurrent injuries, particularly on lameness, proved challenging. This study found that, overall, the short-term outcome in cats with greater trochanteric fracture was good. A very small number of cases experienced complications such as lameness, discomfort and mineralisation of the abductor muscle group. Other injuries can influence the healing process, recovery and prognosis. Therefore, any conclusions regarding the specific outcome of greater trochanteric fracture should be interpreted carefully.

## Conclusions

Greater trochanteric fractures occur in young cats with a mean age of 10.5 months. This fracture was associated with concurrent (in all cases) and multiple (in some cases) orthopaedic injuries, coxofemoral luxation and pelvic/sacral injury being the most common. The ventrodorsal frog-leg radiographic view of the pelvis is the most useful view for the visualisation of greater trochanteric fracture in cats.

Surgical intervention with pins and tension band wire was associated with a good or acceptable short-term outcome in the vast majority of cats. Conclusions regarding the specific outcome of greater trochanteric fracture cannot be accurately commented on, given the presence of concurrent orthopaedic injuries in all cats. However, this is the only study that reports on the imaging and clinical features of cats with a fracture of the greater trochanter.

## References

[1] Hill FW. A survey of bone fractures in the cat. J Small Anim Pract 1977; 18: 457–463. 886835 10.1111/j.1748-5827.1977.tb05912.x

[2] Bookbinder PF Flanders JA. Characteristics of pelvic fracture in the cat. Vet Comp Orthop Traumatol 1992; 37: 122–127.

[3] Guiot LP Dejardin LM. Fracture of the femur. In: Tobias KM Johnston SA (eds). Veterinary surgery: small animal. St Louis, MO: Elsevier, 2013, pp 1019–1071.

[4] DeCamp CE Johnston SA Schaefer SL. Fracture of the femur and patella. In: Brinker, Piermattei and Flo’s handbook of small animal orthopedics and fracture repair. Philadelphia, PA: Saunders Elsevier, 2016, pp 531–536.

[5] Smith RN. Fusion of ossification centres in the cat. J Small Anim Pract 1969; 10: 523–530. 5350530 10.1111/j.1748-5827.1969.tb04071.x

[6] Miranda FG Souza IP Viegas FM , et al. Radiographic study of the development of the pelvis and hip and the femorotibial joints in domestic cats. J Feline Med Surg 2020; 22: 476–483. 31184248 10.1177/1098612X19854809 PMC10814335

[7] Voss K Langley-Hobbs SJ Montavon PM . The femur. In: Feline orthopedic surgery and musculoskeletal disease. Philadelphia, PA: Saunders, 2009, pp 455–473.

[8] Bennett D. Orthopaedic disease affecting the pelvic region of the cat. J Small Anim Pract 1975; 16: 723–738. 1195670 10.1111/j.1748-5827.1975.tb05801.x

[9] Pinna S Cella V. Avulsion of the greater trochanter and craniodorsal luxation of a hip joint in a cat: importance of precise radiographic evaluation. Iran J Vet Res 2014; 15: 413–415. 27175142 PMC4789224

[10] Hulse D Kerwin S Merters D. Fracture of the femur. In: Johnson AL Houlton JEF Vannini R (eds). AO principles of fracture management in the dog and cat. Davos, Switzerland: AO Publishing, 2005, pp 275–285.

[11] Hattori A. Effect of epiphysiodesis of the greater trochanter on the growth of the femur of dogs. J Jap Orthop Assoc 1976; 50: 185–189.

[12] Hauptman J. Effect of osteotomy of the greater trochanter with tension band fixation on femoral conformation in Beagle dogs. Vet Surg 1979; 8: 13–18.

[13] Cook JL Evans R Conzemius MG , et al. Proposed definitions and criteria for reporting time frame, outcome, and complications for clinical orthopedic studies in veterinary medicine. Vet Surg 2010; 39: 905–908. 21133952 10.1111/j.1532-950X.2010.00763.x

[14] Bar-Am Y Pollard RE Kass PH , et al. The diagnostic yield of conventional radiographs and computed tomography in dogs and cats with maxillofacial trauma. Vet Surg 2008; 37: 294–299. 18394078 10.1111/j.1532-950X.2008.00380.x

[15] Piermattei DL Flo GL DeCamp CE . **Approach to the greater trochanter and subtrochanteric region of the femur** . In: Handbook of small animal orthopedics and fracture repair. 4th ed. Philadelphia, PA: Saunders Elsevier, 2004, pp 368–371.

[16] Fischer HR Norton J Kobluk CN , et al. Surgical reduction and stabilization for repair of femoral capital physeal fractures in cats: 13 cases (1998–2002). J Am Vet Med Assoc 2004; 224: 1478–1482. 15124890 10.2460/javma.2004.224.1478

[17] Lambrechts NE Verstraete FJM Sumner-Smith G , et al. Internal fixation of femoral neck fractures in the dog – an in vitro study. Vet Comp Orthop Traumatol 1993; 6: 188–193.

[18] Roberts VJ Meeson RL. Feline femoral fracture fixation: what are the options? J Feline Med Surg 2022; 24: 442–463. 35404170 10.1177/1098612X221090391 PMC11104039

[19] Perez-Aparicio FJ Fjeld O. Femoral neck fractures and capital epiphyseal separations in cats. J Small Anim Pract 1993; 34: 445–449.

[20] Stubbs WP Bloomberg MS Scruggs SL , et al. Effects of prepubertal gonadectomy on physical and behavioral development in cats. J Am Vet Med Assoc 1996; 209: 1864–1867. 8944799

[21] Butts D Smith AJ Bradley K , et al. Comparison of three radiographic assessment methods for detecting slipped capital femoral epiphyses in cats: Klein’s line, modified Klein’s line and the S-sign. J Feline Med Surg 2023; 25. DOI: 10.1177/1098612X231201775. 10.1177/1098612X231201775 PMC10812018 37906175

[22] Carrera I Hammond GJ Sullivan M. Computed tomographic features of incomplete ossification of the canine humeral condyle. Vet Surg 2008; 37: 226–231. 18394068 10.1111/j.1532-950X.2008.00370.x

[23] Guiot LP De Jardin LM . Fractures of the femur. In: Johnston SA Tobias K (eds). Veterinary surgery: small animal. 2nd ed. St. Louis, MO: Elsevier, 2016, pp 1019–1024.

[24] Daly WR. Femoral head and neck fractures in the dog and cat: a review of 115 cases. Vet Surg 2008; 7: 29–38.

[25] DeCamp CE Probst CW Thomas MW. Internal fixation of femoral capital physeal injuries in dogs: 40 cases (1979-1987). J Am Vet Med Assoc 1989; 194: 1750–1754. 2753801

[26] Beale B. Orthopedic clinical techniques femur fracture repair. Clin Tech Small Anim Pract 2004; 19: 134–150. 15712460 10.1053/j.ctsap.2004.09.006

